# Editorial for Special Issue “Dietary and Animal Gut Microbiota”

**DOI:** 10.3390/microorganisms14102234

**Published:** 2026-10-03

**Authors:** Yu Pi

**Affiliations:** Key Laboratory of Feed Biotechnology of Ministry of Agriculture and Rural Affairs, Institute of Feed Research, Chinese Academy of Agricultural Sciences, Beijing 100081, China; piyu@caas.cn

The gut microbiota has long been recognized as a critical “hidden organ” that interacts dynamically with host animals, playing irreplaceable roles in regulating nutrient metabolism, shaping immune function, and maintaining systemic health [1,2]. Over the past decades, advances in high-throughput sequencing, multi-omics integration, and microbial cultivation technologies have deepened our understanding of the composition and function of animal gut microbial communities [3,4]. One of the most well-established core concepts to emerge from this research is the close, bidirectional interaction between diet and the gut microbiota: dietary components are the primary determinant shaping the structure and metabolic activity of gut microbial communities, while the microbiota in turn modulates the host’s nutrient utilization, influences growth performance, and determines disease susceptibility [5,6]. Despite these major research advances, the complex ecological relationships underpinning diet–microbiota–host interactions remain incompletely understood, with critical knowledge gaps persisting across different animal species and diverse production systems covering livestock, aquaculture, and companion animals.

Against this backdrop, this Special Issue aims to collect cutting-edge original research and authoritative reviews that advance our understanding of how diet–gut microbiota interactions shape animal health, growth, and disease resistance, covering a range of key topics including dietary modulation of the gut microbiota, the role of the microbiota in host nutrient metabolism, crosstalk between the microbiota and host immune function, cross-species comparative analyses, and methodological innovations for microbiota research. The collection of work assembled in this Special Issue reflects the diversity and vibrancy of current research in this field: it spans a broad range of animal species from model organisms to production livestock and companion animals, addresses pressing practical questions from improving production efficiency to supporting long-term intestinal health, and explores new mechanistic insights into microbial metabolic regulation.

Dietary functional oligosaccharides as prebiotics are a well-established research direction for regulating animal gut health. One study included in this collection systematically explored the dose-dependent regulatory effect of galacto-oligosaccharides (GOSs) on cecal microbiota and metabolic profiles in healthy mice, confirming that GOS intervention not only reshapes gut microbiota composition, but also significantly regulates key microbial metabolites beyond short-chain fatty acids (SCFAs), including phospholipids and aromatic amino acid metabolites. Of note, high-dose GOS significantly upregulates the production of indole-3-acetic acid—a tryptophan-derived bioactive metabolite with beneficial physiological functions—which expands our mechanistic understanding of how prebiotics improve gut function (Contribution 1). In ruminant production, another study investigated the effects of different supplementation levels of *Bacillus subtilis* on fecal microbiota and metabolites in Leizhou goats, revealing dose-dependent and sex-specific regulatory patterns of microbial composition and metabolism, and providing a practical reference for optimizing the application dose of *Bacillus subtilis* in goat production (Contribution 2). For indigenous pig production, Zhang et al. clarified the sex-specific differences in gut microbial composition and metabolism between the small and large intestines of Jiangshan Black pigs, finding notable sex discrepancies in the enrichment of microbial taxa and the production of SCFAs across different intestinal segments, which provides a basis for precise sex-specific regulation of pig gut microbiota (Contribution 3).

Against the core industry goal of improving feed utilization efficiency and reducing environmental footprints in ruminant livestock, multiple original studies in this Special Issue explored the methane reduction potential of seaweed-derived additives and the regulatory effect of functional dietary components on rumen development and health from complementary perspectives. Focusing on the key priority of enteric methane reduction for sustainable ruminant production, one in vitro study systematically evaluated the anti-methanogenic potential of five Indian coastal seaweeds and optimized the effective addition levels of the most promising strains for straw–concentrate mixed diets, confirming that these seaweeds deliver significant methane reduction without impairing normal rumen fermentation. The study found that adding 3–5% dry matter (DM) *Kappaphycus alvarezii* reduced methane production by 31–42%, while adding 4–5% DM *Sargassum wightii* reduced methane emission by 36–48%. Further metagenomic analysis revealed that both seaweeds significantly reduced the counts of total protozoa and *Entodinomorphs* and increased the abundance of sulfate-reducing microbes and low-abundance methanogens, but did not alter the dominant position of core microbial phyla (*Bacteroidota*, *Bacillota*, *Pseudomonadota*, etc.) or *Methanobrevibacter*—the dominant methanogen, which accounts for ~79% of the archaea community. The methane reduction effect does not stem from the disruption of basal rumen fermentation, confirming the safety and effectiveness of these two seaweeds as methane-reducing feed additives and laying a foundation for subsequent in vivo validation (Contribution 6). Another methane reduction study tested the application potential of low-cost marine algal biowaste *Padina gymnospora* by replacing wheat bran in the concentrate diet of growing sheep. It found that adding 5% *Padina gymnospora* biowaste (of total diet DM) did not affect nutrient intake, digestibility, or average daily gain (ADG) of growing sheep, but significantly reduced daily enteric methane emission by 28.4% and energy loss as a percentage of gross energy by 23.4%. Combined rumen metagenomic analysis attributed this methane reduction effect to the significant decrease in total protozoa and *holotrichs* abundance and the significant increase in the abundance of sulfate-reducing bacteria *Desulfovibrio* induced by biowaste addition. This confirms the application potential of low-cost marine algal biowaste as a dietary methane-reducing additive for ruminants and provides a practical alternative strategy for green emission reduction in sheep production (Contribution 5).

To improve rumen development and growth performance of fattening Hu sheep, a study evaluated prickly ash seeds (PASs)—a common plant feedstuff rich in bioactive components—and revealed that adding 3% and 6% PAS to the basal diet improved ADG, increased feed conversion ratio (FCR), and significantly increased the height and width of rumen papilla. Combined metagenomic and metabolomic analysis showed that PAS supplementation increased the abundance of Clostridiales and Bacteroidales, reduced the abundance of *Prevotella*, *Butyrivibrio*, and *Methanococcus*, and promoted the accumulation of growth-related metabolites such as serotonin, L-isoleucine, and L-valine. Further correlation analysis confirmed that these regulatory changes in microbiota and metabolites are closely associated with improvements in rumen epithelial development and growth performance, indicating that PAS can improve sheep growth performance and disease resistance by reshaping the rumen microbiota and metabolome, and providing a new natural feed additive option for efficient Hu sheep production (Contribution 8). For indigenous Tan sheep, a dominant local mutton breed, the FCR is a key economic trait that directly determines production benefit. To clarify how multi-site gastrointestinal microbiota affects FCR, one study systematically integrated 16S rRNA sequencing, untargeted metabolomics, and RT-qPCR to compare differences in microbial community structure across the rumen, cecum, and rectum, as well as differences in rumen metabolites, between high-FCR (low feed efficiency) and low-FCR (high feed efficiency) Tan sheep. This work not only identified differential microbial taxa significantly correlated with FCR across different intestinal sites, but also revealed a consistent co-association pattern between core differential microbes in the rumen and key differential microbes in the hindgut, linking core differential microbes to their corresponding differential rumen metabolites. Specifically, the study found that core differential taxa unclassified_f__Selenomonadaceae, Blvii28_wastewater-sludge_group, and *Papillibacter* in the rumen were positively correlated with differential metabolites such as L-tryptophan, Etiocholanolone glucuronide, and Icilin, and also had significant positive co-occurrence relationships with FCR-related differential taxa in the cecum and rectum. This work preliminarily revealed the cooperative mechanism of multi-site gastrointestinal microbiota in regulating feed digestion and absorption in Tan sheep, and provides an important foundational basis for subsequent microbial improvement of ruminant feed efficiency (Contribution 4).

For non-ruminant poultry production, this Special Issue also expands our understanding of how novel dietary additives regulate intestinal homeostasis. One study evaluated the effect of copper–carbon nano-composites (NCCC), an emerging novel trace element additive, on the intestinal luminal microecosystem and mucosal homeostasis of yellow-feather broilers. The results showed that NCCC supplementation has only subtle effects on the cecal microbiota structure of broilers: only low-dose (50 mg/kg) and high-dose (200 mg/kg) NCCC reduced the relative abundance of a small number of specific taxa (*Ruminococcus torques* group and norank Clostridia UCG-014, respectively) without altering overall microbial composition. By contrast, NCCC significantly improved intestinal mucosal function: it linearly increased jejunal villus height, optimized the expression of tight junction protein genes, upregulated anti-inflammatory cytokine levels, and improved intestinal mucosal antioxidant capacity. Overall, 50 mg/kg of dietary NCCC supplementation is recommended to maintain intestinal homeostasis in yellow-feather broilers, providing a basis for the application of this novel nano-trace element additive in poultry production (Contribution 7).

In swine production, this Special Issue includes a targeted review that addresses key demand for safe, sustainable feed additive strategies for pig production. The review by Fan et al. systematically consolidates current research progress on the utilization of yeast and its derivatives (including yeast extract and yeast cell wall) as environmentally friendly, safe feed additives in swine production. As a versatile fermentative microorganism, yeast produces a variety of bioactive components including amino acids, vitamins, enzymes, and growth factors, which collectively contribute to improved growth performance, enhanced immune function, and better overall health in pigs. This review synthesizes the established biological functions of yeast and yeast derivatives and clarifies their practical application value and industrial implications for modern swine production (Contribution 11).

Beyond nutritional strategies for production performance, the Special Issue also covers fundamental microbial ecology across animal taxa. The review by Gul et al. provides a comprehensive and in-depth exploration of the understudied phenomenon of parental coprophagy in wild birds and investigates the key role of microbiota in this behavior. The review systematically analyzes the composition and dynamic changes in microbiota in avian feces, summarizes the dietary, environmental, and genetic factors that shape microbial diversity, and synthesizes multiple existing hypotheses for the occurrence of parental coprophagy—a behavior widespread across many bird species, especially herbivorous and passerine birds. The review highlights the emerging hypothesis that parental coprophagy acts as a mechanism for microbial transmission from nestlings to adult parents, which may further shape the health and fitness of adult breeders. It also systematically sorts out current research gaps and academic debates: these include unclear onset timing of coprophagy, unknown long-term effects on both parent and offspring fitness, unresolved nutritional significance of nestling feces consumption, unquantified risks of pathogen transmission, and underexplored ecological and evolutionary drivers of this behavior. By synthesizing existing evidence and identifying undressed research priorities, this review points out key directions for future research to reveal the ecological and evolutionary significance of parental coprophagy in wild birds, which will advance avian biology and provide scientific support for the conservation of migratory bird populations and their complex ecosystems (Contribution 12).

In the field of feed protein processing, the review by Mulalapele et al. provides a critical synthesis of the biochemical transformation from raw soybean meal (SBM) to fermented soybean meal (FSBM), with a specific focus on the synergistic mechanisms of fungal–bacterial co-fermentation (Contribution 9). The review confirms that the efficacy of FSBM is primarily driven by microbial proteolysis of glycinin into low-molecular-weight bioactive peptides (<1000 Da). These peptides are the core drivers of improved intestinal morphology (increased villus height) and gut microbiota modulation, providing a mechanistic basis for the documented probiotic effects of FSBM. Furthermore, the review verifies that the 5–10% improvement in FCR observed in swine production mathematically offsets the additional processing cost of fermentation. However, it also highlights critical gaps in the standardization of solid-state fermentation (SSF) protocols, especially regarding the selection of fungal (*Aspergillus*) and bacterial (*Bacillus* or *Lactobacillus*) strains, whose distinct metabolic pathways generate considerable diversity in the functional profile of the final FSBM product. This review clarifies the current state of knowledge and unaddressed priorities for FSBM application in animal husbandry, offering valuable guidance for future industrial development.

For companion animals, the review by Yang et al. condenses current evidence on the bidirectional diet–gut microbiota connection and the application of nutritional therapy for gastrointestinal, metabolic, renal, hepatic, and immune-mediated disorders in dogs and cats (Contribution 10). It summarizes that protein-based dietary interventions (including high-protein or hydrolyzed protein diets), combined with omega-3 fatty acids, fermentable fiber, and probiotics, can positively shape microbial composition, stimulate SCFA synthesis, and enhance intestinal barrier function. Conversely, excess fat and refined carbohydrates may induce dysbiosis, inflammation, and metabolic imbalance. Numerous studies have demonstrated that therapeutic nutrition—such as low-protein renoprotective diets, hepatoprotective antioxidant regimens, and allergen-elimination diets—holds enormous clinical potential for disease management. In addition, fecal microbiota transplantation (FMT) can be used as an adjunct therapy for refractory gastrointestinal diseases. Despite these advances, limitations remain in terms of protocol standardization, study duration, and species-specific data, especially for cats. This review underscores dietary modification as a clinically actionable tool for microbiota-targeted therapy, and calls for integrative multi-omics research to translate microbiome modulation into precision nutrition for companion animals.

The eight original experimental studies included in this Special Issue cover a wide range of animal species and core scientific questions in the field of dietary regulation of animal gut microbiota, and together they provide new data, new insights, and new practical strategies for this research direction. The four reviews included in this collection systematically sort out emerging frontiers and key unresolved issues spanning yeast additive application in swine production, avian microbial behavioral ecology, fermentation-modified feed proteins, and companion animal microbiota-targeted nutritional therapy, further enriching the breadth and depth of this Special Issue.

We hope that this Special Issue will stimulate new cross-institutional and cross-disciplinary research collaborations, advance our understanding of diet–gut microbiota interactions in animals, and ultimately support the development of sustainable, healthy, and efficient animal production systems that benefit both animal welfare and human food security.
